# Supplementary material for: SENSITIVITY BASED MODEL AGNOSTIC SCALABLE EXPLANATIONS OF DEEP LEARNING
Source: bioRxiv. 2025 Mar 7:2025.02.21.639516. Preprint. [Version 2] doi: 10.1101/2025.02.21.639516 (PMC11908179; doi:10.1101/2025.02.21.639516)
Supplement: Supplement 2 [file media-2.pdf]

Model/datase	F1-score on test data
XOR	0.99
orange_skin	0.97
nonlinear_a[illegible]	0.99
switch	0.98

model	top-k	median accuracy (%)		accuracy differential (%)	mean time taken (sec)		speed-up factor	CQD (lower is better)	
		SHAP	SensX		SHAP	SensX		SHAP	SensX
XOR	1	99.2	100	**+0.8**	296	14	**21**	0.0015	**0**
	2	97.9	100	**+2.1**	2190	14	**156**	0.002	**0**
orange_skin	1	100	100		2206	14	**158**	0	0
	2	99.8	100	**+0.2**	2206	14	**158**	0.0005	**0**
	3	98.7	100	**+1.3**	2206	14	**158**	0.001	**0**
	4	89	99.8	**+10.8**	2206	14	**158**	0.0039	**0**
nonlinear_additive	1	100	100		2210	14	**158**	0	0
	2	99.7	100	**+0.3**	2210	14	**158**	0.001	**0**
	3	96	100	**+4.0**	2210	14	**158**	0.0052	**0**
	4	72	94.3	**+22.3**	2210	17	**130**	0.0254	**0.0085**
switch	1	88.5	99.9	**+11.5**	296	14	**21**	0.0479	**0**
	2	50.5	99.5	**+49.0**	296	14	**21**	0.1089	**0.0005**
	3	16.3	67.6	**+51.3**	296	14	**21**	0.2183	**0.0037**
	4	7.2	59.2	**+52.0**	2218	14	**158**	0.049	**0.0025**
	5	2.4	12	**+9.6**	1123	9	**125**	0.125	**0.0661**

Eyeglasses-ViT

	Train	Test	Total
FALSE	151525	37881	189406
TRUE	10555	2638	13193
% TRUE	6.51221619	6.51052593	6.51187814

Smiling-ViT

	Train	Test	Total
FALSE	83944	20986	104930
TRUE	78136	19533	97669
% TRUE	48.2082922	48.207014	48.2080366

Models	F1-score on test
Smiling	0.91
Eyeglasses	0.92

cell type	F1 score test data
lveolar macrophage	0.97572337
natural killer	0.9717783
CD4 T	0.93786632
CD8 T	0.93556662
Classical monocytes	0.94307875
elicited macrophage	0.89077587
Non-classical monocytes	0.92085559
pulmonary alveolar type 1	0.97926925
pulmonary alveolar type 2	0.99042482
capillary endothelial	0.97593563
ciliated columnar tracheobronchial tree	0.99126953
CD1c-pos	0.91643286
nasal mucosa goblet	0.95301045
vein endothelial cell	0.96219065
respiratory basal	0.98048556
mast	0.99329033
pulmonary artery endothelial	0.94153758
alveolar type 1 fibroblasts	0.98639859
adventitial fibroblasts	0.97847957
club	0.92358233
B	0.99393129

cell type	perturbation factor		top k SensX perturbed	
	median	CQD	median	CQD
mast	0.24	0.14	566	0.33
club	0.09	0.18	284	0
B	0.25	0.14	284	0.33
alveolar macrophage	0.14	0.17	284	0
natural killer	0.14	0.24	284	0
CD4 T	0.09	0.26	284	0
CD8 T	0.12	0.17	284	0
classical monocytes	0.13	0.2	284	0
elicited macrophage	0.08	0.25	284	0
non-classical monocytes	0.13	0.25	284	0
pulmonary alveolar type 1	0.21	0.25	284	0
pulmonary alveolar type 2	0.2	0.17	284	0
capillary endothelial	0.17	0.21	284	0
ciliated columnar tracheobronchial tree	0.28	0.14	284	0.33
CD1c-pos	0.1	0.3	284	0
nasal mucosa goblet	0.13	0.2	284	0
vein endothelial cell	0.23	0.17	284	0
respiratory basal	0.14	0.26	284	0
pulmonary artery endothelial	0.15	0.23	284	0
alveolar type 1 fibroblasts	0.25	0.19	284	0
adventitial fibroblasts	0.2	0.21	284	0

Model	Significant perturbations				SensX
	n_s	Delta	tau_a	tau_r	n_w
Synthetic	2000	lin(0.02, 1, 50)	0.1	0.1	multiple
ViT	1000	lin(0.02, 1, 50)	0.1	0.1	500
single-cell	1000	geom(0.0001, 1, 50)	0.1	0.1	200
